# Sensory Processing Patterns and Sleep Quality in Primary School Children

**Published:** 2020

**Authors:** Samira RAJAEI, Minoo KALANTARI, Zahra PASHAZADEH AZARI, Seyed Mehdi TABATABAEE, Winnie Dunn

**Affiliations:** 1 Physiotherapy Research Center, School of Rehabilitation, Shahid Beheshti University of Medical Sciences, Tehran, Iran; 2 Department of Occupational Therapy, School of Rehabilitation,Shahid Beheshti University of Medical Sciences, Tehran, Iran; 3 Department of Occupational Therapy, University of Missouri, Columbia, USA

**Keywords:** Sleep, sensory processing patterns, sleep quality, primary school children

## Abstract

**Objectives:**

Sensory processing and sleep quality affect children's academic performance and their quality of life. This study aimed to investigate the relationship between sensory processing patterns and sleep quality in primary school children.

**Materials & Methods:**

In this cross-sectional study, 231 primary school students aged 7 to 12 years old (133 girls and 98 boys, the mean age of 8.68±1.51) who were studying in schools in Tehran were randomly selected through cluster sampling. The researchers distributed a questionnaire on children's sleep habits to assess the quality of sleep and a sensory profile questionnaire to assess the sensory processing patterns (avoidance, sensitivity, seeking, and registration) among the students.

**Results:**

In this study, we found a meaningful moderate relationship between sensory processing patterns and overall scores of sleep habits (p <0.001). Moreover, each of the sensory processing patterns had a negative relationship with areas of sleep habits (p = 0.005). There was also a significant difference between children who had more challenges with sleep maintenance and children with normal sleep patterns in sensory processing; mean differences were significant in all the four sensory quadrants (registration, seeking, sensitivity, and avoiding) (p <0.001).

**Conclusion:**

The sensory processing patterns are moderately correlated with sleep habits in primary school children. Occupational therapists and other specialists working in the field of children's sleep should consider the relationship between sensory challenges and sleep habits while making decisions about sensory challenges and sleep problems. Better sleep may occur with attention to sensory needs in sleep routines. Better sleep may lead to improved quality of life in families and enhanced student performance at school.

## Introduction

About 43% of school age children experience disruptions in sleep (1). A sleep problem is the fifth cause of referrals to physicians (2). According to the *occupational therapy practice framework* (OTPF), sleep is one of the areas of activity that supports active and healthy participation in other areas of life (3). Sleep is also considered a necessary prerequisite for children's function (4). Studies on children have indicated that poor sleep can lead to obesity (5, 6), depression symptoms (7), attention deficit-hyperactivity disorder (8), and poor neuro-behavior function (9). Moreover, researchers have shown that sleeping problems at the age of eight predict depression at the age of 10 (10). Poor sleep may impair memory, concentration, and interpersonal relationships. Generally, poor sleep may result in poor quality of life, as well as in poor mental and physical well-being. Therefore, low sleep quality results in increased health care costs in the community (11). People with low sleep quality appear to show signs of high arousal levels during sleep. The level of arousal is associated with both low sleep quality and sensory processing, which can be due to a lack of inhibition in the central nervous system (11-14).

Recent research has shown a relationship between sleep problems and sensory processing patterns. Sensory processing is the internal process of the central nervous system for receiving, organizing, and understanding sensory inputs (15). Differences in expected sensory processing patterns may be related to regulating and organizing the type and intensity of sensory inputs for adaptation to environmental requirements (16). 

Among children without any disabilities, 5-10% have differences in sensory patterns (17). According to Dunn's sensory processing framework, a relationship exists between neural thresholds and behavioral responses (18). According to this framework, there are four sensory processing patterns: registration, sensation seeking, sensory sensitivity, and sensation avoiding. Based on these four patterns, it is possible to interpret the child's behavior from a sensory point of view (19).

The relationship between sensory processing and sleep has been investigated in children with autism, attention deficit hyperactivity disorder, and alcoholic fetal syndrome, as well as in normal school children, infants, and toddlers (20-24). In 2011, Reynold et al. found a relationship between the sensory processing patterns and sleep behaviors in autistic and typical children. According to the results, sensory avoiding behaviors were highly correlated with sleep problems in autistic children, while sleep problems were strongly correlated with all the four sensory processing quadrants in typical children (20). The results also revealed that sleep problems were associated with sensory processing deficits in children with alcoholic fetal syndrome (21, 22), and that increased tactile sensitivity was related to sleep problems among normal school children (23). Vasak et al. (2015) reported correlations between increased seeking and shorter daytime sleep duration as well as between increased sensitivity and longer time to settle to sleep in typically developing infants and toddlers (24). 

Research has not been so far conducted in Iran on patterns of sensory processing, as well as on habits and quality of sleep. Understanding what both improves and interferes with sleep is critical to occupational therapists because it is an area of occupation. Moreover, few studies have examined the relationship between all four sensory patterns and sleep quality in primary school students. This study may help to alter and improve management strategies in sleep problems, and can be beneficial in improving daily performance among primary school students. This study aimed to investigate the relationship between the sensory processing patterns and sleep quality in primary school students. It also aimed to investigate differences in sensory processing scores between children with normal sleep and those with a sleep disorder.

## Materials

**Study design:**

This descriptive study was carried out using random cluster sampling.

**Participants:**

At first, five districts of Tehran were selected (22, 5, 13, 14, and 8) randomly. Then, from each district, a school was selected, and after coordination with the schools, all parents of children aged 7 to 12 years old were invited to a free workshop at the school for participating in the study. Workshops were about students' sleep problems, which lasted about 3 hours. We distributed a demographic questionnaire and written consent forms among 300 parents. Our exclusion criteria were the use of drugs affecting sleep rhythm (clonidine, melatonin, atomoxetine, and ritalin if used later than 6 PM), surgeries due to respiratory-sensory-sleep problems, respiratory diseases, and psychiatric disorders. A total of 252 parents whose children participated in the study signed the consent form. Then, the Children's Sleep Habit Questionnaire (CSHQ) and the Sensory Profile Questionnaire (SPQ) were given to the parents. Twenty-one copies of the questionnaires were filled out incompletely, and finally, we collected 231 completed copies.

All parents signed written consent. The study was approved by the Ethics Committee of Shahid Beheshti University of Medical Sciences with the code IR.SBMU.RETECH.REC.1396.640.

**Tools:**

The Demographic Information Questionnaire:

The demographic information questionnaire includes items on age, gender, parental education, consumer medications, respiratory and sensory system surgeries, respiratory diseases, and psychiatric problems, which were answered by the parents.

The Sleep Habit Questionnaire:

The Persian version of CSHQ was used to evaluate sleep habits of children aged 7 to 12 years old. The parents completed CSHQ. It contains 33 items and eight subscales: bedtime resistance, sleep onset delay, sleep duration, sleep anxiety, Night wakings, parasomnias, sleep disordered breathing, and daytime sleepiness. Scores range is from 33 to 66, and the cut-off point> 42 indicated poor sleep habits in children, and the higher the score, the worse the sleep habits. The items were scored based on a three-point Likert scale: if the behavior occurs 5 to 7 times per week, the option "usually" (score 3), 2 to 4 times per week, the option "sometimes" (score 2), and 0 to 1 time per week (score 1), the option "rarely". The reliability of the Persian version of the questionnaire was measured using Cronbach's alpha (0.80 for the entire questionnaire). The range was from 0.4 to 0.86 for convergence validity and from 0.006 to 0.66 for divergence validity, and good reliability was among Iranian children (25).

The Sensory Profile Questionnaire:

SPQ inquires about the frequency of behaviors related to sensory processing (26). The questionnaire contains 125 items and is completed by parents. Items of the questionnaire are scored on a five-point Likert scale ranging from always=1 to never=5 based on the repetition frequency of the behavior. Lower scores in each section represent undesirable sensory behaviors. Scores can be divided into four sections or quadrants based on the sensory processing model. Each of the four quadrants represents the interaction between the individual's neuronal threshold and behavioral strategies used to respond to sensory information. The four quadrants are registration, seeking, sensitivity, and avoiding. Seeking is a model of sensory processing, in which a high neurological threshold as well as self-regulating strategies and responses are active. In registration, a high neurological threshold as well as self-regulation strategies and responses are inactive and passive. In sensory avoidance, a low neurological threshold, along with self-regulatory strategies and responses, is active. Sensory sensitivity is a pattern of sensory processing characterized by a low neurological threshold as well as self-regulating strategies and inactive responses (27).

Five categories and the cut-off score for the quadrants, which represent a set of capabilities for sensory integration, are much less than others (more than 2 standard deviations above the average), less than others (between 1 and 2 standard deviations above the average), much like others (about 1 standard deviation from the average), more than others (between 1 and 2 standard deviations below the average), and much more than others (more than 2 standard deviations below the average) (28). In this study, we merged the categories one and two (much less than others and less than others) and the categories four and five (more and much more than others) based on the cut-off points. Thus, three categories were formed. The sensory profile was normed on 1000 children without disabilities and 150 children with disabilities. Reliability includes internal consistency estimates (range = 0.47–0.91) and the standard error of measurement (range = 1.0–2.8). **The value of Cronbach’s **alpha **for each of the various sections ranged from 0.47 to 0.91 (**28**). **In Iran, the Cronbach's alpha coefficient for all the parts is between 0.45 and 0.97 (29).

**Data analysis:**

We used the Spearman correlation coefficient to examine the relationship between the quadrant scores of the sensory profile with the scores of the different sections and the total score of sleep habits. To compare the sensory quadrants, the children were divided into two groups of normal sleep and sleep disorders based on the cut-off point> 42, which indicated sleep disorders. In order to examine the difference in the three parts, as well as others, less and much less than others, and more and much more than others, chi-square (χ2) was used in the two groups of children. Regarding the non-normal distribution of the variables, the Mann-Whitney U test was used to examine the difference between the sensory quadrants and to compare means in the two groups.

## RESULTS

The demographic information of the 231 children was analyzed and is summarized in Table 1. According to Table 2, there was a moderate negative relationship between the total sleep score and sensory processing patterns in each of the four quadrants (p <0.001). 

The sensory processing patterns in all the three sections were significantly different in the both groups based on the thex2 test (Table 3). In the Mann-Whitney U test, the mean was significantly different in all the four quadrants at p <0.001 (Table 4).

**Table 1:** The demographic information of the participants

**98(42%)**	Male	Gender
**133(58%)**	Female	
**8.68** **±** **1.51**	Mean	age
**115(49.7%)**	Diploma and lower	parental education
**67(29%)**	Bachelor	
**14(6.1%)**	Masters degree and higher	
**35(15.2%)**	Unknown	
**57(24.7%)**	Normal^*^	sleep
**174(75.3%)**	Disorder ^**^	

**(>42)*(≤42)

**Table 2:** The correlation coefficient between sleep habits and the sensory processing patterns

		Bedtime Resistance	Sleep Onset Delay	Sleep Duration	Sleep Anxiety	Night Wakings	Parasomnias	Sleep Disordered Breathing	Daytime Sleepiness	Sleep Total
Poor sensory registration	**R**	-0.112 NS	-0.056 NS	-0.149*	-0.204**	-0.112 NS	-0.322***	-0.211**	-0.284***	-0.334***
Sensory seeking	**R**	-0.155*	-0.150*	-0.225***	-0.267***	-0.190**	-0.308***	-0.214***	-0.271***	-0.381***
Sensory sensivity	**R**	-0.209***	-0.105 NS	-0.161*	-0.243***	-0.198**	-0.311***	-0.258***	-0.277***	-0.389***
Sensory avoiding	**R**	-0.202**	-0.088 NS	-0.171**	-0.182**	-0.202**	-0.311***	-0.200**	-0.241***	-0.360***

Note. NS = not significant.*p < 0.05.**p < 0.01.***p < 0.001.

**Table 3:** Differences between sleep disorders and normal sleep in the three groups

**Sig. (2-sided)**	Chi-Square(x^2^)	Sleep		AASP* quadrants	
		Disorder	Normal		
**0.012**	8.89	31	19	Less and much less than others	registration
		71	25	Like others	
		72	13	More and much more than others	
**0.03**	6.99	8	6	Less and much less than others	seeking
		76	32	Like others	
		90	19	More and much more than others	
**0.001**	15.05	6	8	Less and much less than others	sensivity
		77	33	Like others	
		91	16	More and much more than others	
**0.001**	14.00	21	15	Less and much less than others	avoiding
		87	34	Like others	
		66	8	More and much more than others	

* AASP=Adolescent/Adult Sensory Profile

**Table 4:** Comparison of sensory processing means in the two groups

Sensory processing patterns	Normal sleep mean		Sleep disorder mean		Difference of means	z	P
Registration		68.67		63.39	5.28	-3.585	<0.0001
Seeking		106.91		99.25	7.66	-3.578	<0.0001
Sensivity		85.46		78.15	7.31	-3.880	<0.0001
Avoiding		125.51		116.13	9.38	-3.900	<0.0001

## Discussion

This study aimed to investigate the relationship between the sensory processing patterns and sleep habits in primary school children. We found a significant correlation between the sensory processing patterns and the overall sleep habit score. Accordingly, sensitivity, avoidance, registration, and seeking were associated with low scores in children’s sleep habits. According to Dunn, the sensory processing patterns are biological phenomena that persist throughout life (30) and, as a result, are related to the quality of sleep (11).

Various studies have shown that people with insomnia are more irritable than those who have better sleep. They are not able to modulate and regulate sensory stimuli (14, 31, 32). According to Milner et al.’s findings, sensory sensitivity is present in people with poor sleep (32).

According to our findings, there was a significant linear correlation between sensory sensitivity and most sleep habits. It means that children with more sensory sensitivity showed greater resistance to sleep. Night wakings, parasomnia, sleep anxiety, sleep disordered breathing, increased duration of sleep, and daytime sleepiness were more common among our subjects. In other words, sleep disturbance was generally high in this population. This finding is consistent with the study by Vasak et al. (2015) in children and infants, showing that children with a sensory sensitivity needed more time to go and stay in bed than those with normal function (24).

 Further, Shochat et al. (2009) reported a significant negative correlation between the sensory sensitivity patterns and sleep habits in normal primary school children (23). According to their study, sensory sensitivity increased by decreasing sleep quality in children with atopic dermatitis (AD) aged 4 to 10 years old. Increased auditory and visual sensitivity was reported to be associated with increased sleep anxiety. In addition, increasing visual sensitivity led to an increase in parasomnias in these children (33).

Children with a low threshold of neurological or increased sensitivity to environmental stimuli are at high levels of excitability, anxiety, and impulsivity (34), causing sleep problems and low sleep quality. The low neural threshold leads to avoidant behaviors, which may be one of the reasons for reduced nightly sleep quality and sleep disorders (24). Children who are sensory sensitive actively avoid and distract sensory environments that annoy them (35). In our study, increased sensory avoidance was associated with an increase in night wakings, parasomnias, sleep disordered breathing, sleep anxiety, and sleep habits in general. In neonates and toddlers (0 to 36 months), sensory avoidance did not show a significant relationship with sleep quality (24).

In registration, the high neural threshold and self-regulatory strategies are inactive, and one does not record enough sensory information from the environment. In our study, a poor sensory registration in the children was correlated with an increase in sleep anxiety, sleep disordered breathing, parasomnias, daytime sleepiness, and generally, poor sleep habits, which are in line with the results of Wengel et al. (2011) about children with fetal alcohol spectrum disorder (FASD). Among children with FASD, poor sensory registration patterns are associated with parasomnia, and a weaker sensory registration increases parasomnia (21). Our findings are unlike the results about infants and toddlers (0 to 36 months), showing that no meaningful relationship existed between poor sensory registration and sleep quality (24). Normally, until the age of 2, the duration of sleep increases, and nightly waking and daily sleep are reduced. Thus, a poor sensory registration may have low effect on sleep habits in younger children. A high neurological threshold leads to sensory seeking in children. In our study, children who were more sensory seeking had worse sleep habits and more sleep-related problems. These results agree with Shochat's findings, stating that increased sensory seeking was associated with increased sleep disturbance (bad sleep habits) in normal school children (23). Moreover, among children with FASD, there was a significant correlation between sensory seeking and daily sleep time (22). Additionally, in the study of Shani-adir, increased sensory seeking was associated with the duration of sleep in children with AD (33).

In all the four quadrants, people with sleep disorder were in the more than others group, indicating that a large number of individuals with sleep disorder had a poorer sensory registration, poorer sensory seeking, greater sensory sensitivity, and a more severe sensory avoidance compared to those with normal sleep. The difference in mean values of the four quadrant sensory status ​​was significant in the both groups.

**Limitations and future research:**

One of the limitations of the study was the large number of items in the questionnaires, causing the parents to feel exhausted, leave the questionnaires incomplete in some cases, and soon withdraw from the study.

In future research, actigraphy is recommended to be used to study sleep among children. It is better to examine the relationship between different sensory parts such as smell, hearing, touch, and taste with sleep habits, and to study the effect of each of them on sleep habits in Iranian school children. In addition, probable differences should be examined in the sensory processing patterns of normal children with developmental disorders.

**In conclusion,**

there is a moderate correlation between the high neurological threshold (poor sensory registration and sensory seeking) and the low neurological threshold (sensory sensitivity and sensory avoiding) with sleep quality and habits in primary school children in Iran. These findings suggest that children with sleep problems should be precisely evaluated in terms of the sensory processing patterns to improve their performance and increase their quality of life.
